# Variable Bessel Beam Profiles Generated through Refraction by Liquid Media

**DOI:** 10.3390/mi14081609

**Published:** 2023-08-15

**Authors:** Dina C. Palangyos, Raphael A. Guerrero

**Affiliations:** 1 Department of Physics, School of Science and Engineering, Ateneo de Manila University, Loyola Heights, Quezon City 1108, Philippines; dpalangyos.urdaneta@psu.edu.ph; 2 Math and Natural Sciences Department, College of Computing, Pangasinan State University San Vicente, Urdaneta City 2428, Philippines

**Keywords:** Bessel beam, axicon, core diameter, refraction, liquids

## Abstract

Various methods have been employed to produce Bessel beams (BBs), with axicon-based techniques remaining the most efficient. Among the limitations of axicons are manufacturing defects such as oblate tips and difficulty in tuning the generated BBs. In this work, we combine the effect of a blunt-tip axicon with refraction using various combinations of liquid media to generate variable BB intensity profiles. The output BBs from the axicon are made to pass through a custom-built fluid chamber and magnified using a telescope system. When traversing an empty chamber, the Bessel beam core diameter is measured to be 773.8 µm at propagation distance *z*’ = 30 cm. The core diameter increases as the beam passes through a chamber containing different liquids as a result of an effective axicon–telescope distance produced by the indices of refraction of the pertinent fluids. Bessel beams modified by the fluid chamber maintain the properties of non-diffraction and self-healing.

## 1. Introduction

Bessel beams (BBs) have proven to be of extensive utility in numerous and diverse optical systems. The usefulness of this class of beams, which are physical approximations of diffraction-free solutions to the Helmholtz wave equation [1,2,3], stems from inherent propagation invariance [4,5] and the ability to self-heal after encountering an obstacle [5,6,7]. The familiar zero-order Bessel beam manifests an intensity profile consisting of a bright central maximum surrounded by concentric rings [3]. Current applications of BBs include industrial glass processing [8,9], optical trapping [3], optical micromanipulation [8,10,11], superior imaging systems [8], optical coherence tomography [8,12], information encryption in optical communications, underwater optical communications, long-distance free-space communications, and sharp focusing [8]. The experimental demonstration of a Bessel beam was first achieved with an annular slit placed on the focal plane of a lens [13], but this early technique had the drawback of low power efficiency. Higher efficiency in Bessel beam generation became available with the use of a conical lens or axicon, with the axicon apex angle determining both core radius r_o_ and maximum propagation distance *z_max_* [3,9,14,15,16].

The ability to efficiently manipulate the intensity profile and propagation behavior of BBs extends the experimental range and versatility of any system employing such beams. Tuning the apex angle of an axicon would lead to a variable Bessel beam profile, but lenses are typically fabricated from rigid materials and have fixed geometries [5,15,16,17]. Modulation of the on-axis intensity profile, as well as the core radius of BBs, has been observed but with axicons having rounded tips [9,16,17,18,19,20,21,22]. Oblate axicon tips are unavoidable deviations from the ideal conical shape, resulting from limitations of the manufacturing process. Previous reports on modifying BB intensity profiles involve combinations of axicons and telescope systems [5,10], varying the distance between the axicon and telescope [21] or between the axicon and an imaging lens [23], and employing a spatial light modulator (SLM) to recreate the action of a tunable axicon with the generated BBs having properties surpassing those produced by real axicons [24].

A particularly promising approach is based on fluidic systems. Bessel beams were produced with a liquid chamber molded from an axicon, and core diameters were varied by filling the chamber with sucrose solutions at different concentrations [15]. Different BB profiles were also generated by immersing a large-angle axicon in an index-matching liquid [18]. An annular slit–lens technique was used to generate Bessel beam profiles that were altered after propagating through layers of different fluids [25].

In this paper, we discuss the successful implementation of a liquid-based technique for tailoring zero-order Bessel beam intensity profiles. Our method takes advantage of the distance-dependent output of a blunt-tip axicon by varying the effective propagation distances of Bessel beams with a refracting fluid chamber. We are able to describe the experimental results with a simple geometric framework. This approach to producing variable Bessel beams is reported here for the first time, to the best of our knowledge. An advantage of the scheme reported here is that no physical movement of optical elements is involved in modifying the output BBs. Our fluid-based system is significantly less expensive than a spatial light modulator and avoids issues with high laser powers that could damage the liquid crystal elements of SLMs [24,26]. Our findings show promise in extending the useful range of Bessel beam core diameters and propagation distances, particularly in high-intensity applications where axicons are the most efficient means of generating BBs [3,9,14,15,16]. Added flexibility in BB geometry and propagation behavior would be beneficial to optical micromanipulation and material processing.

## 2. Theoretical Framework

**Figure 1:**
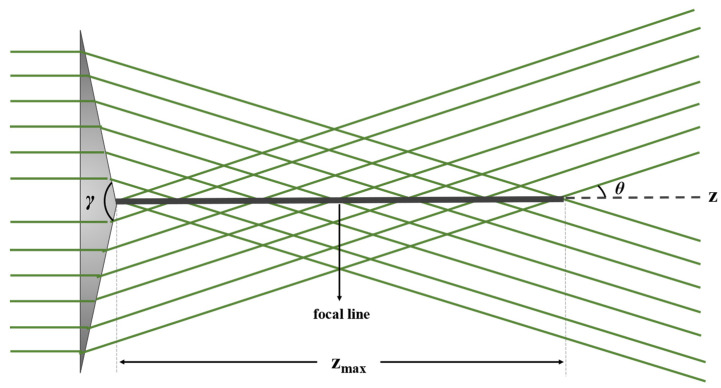
Refracting axicon with apex angle γ: interference of waves along the focal line leads to a Bessel beam propagating for distance *z_max_*.

A refracting axicon, as seen in Figure 1, is a conical lens typically characterized by refractive index *n* and apex angle *γ*, surrounded by a medium with refractive index *n*_0_. We consider an axicon illuminated by a collection of rays that are propagating parallel to the *z*-axis. These rays are refracted at the conical surface toward the axis of propagation, at the same angle *θ*, creating a focal line. *θ* can be derived from Snell’s law [21]:

(1) $$\theta =\arcsin\left(\frac{n}{n_{0}}\cos\frac{\gamma }{2}\right)+\frac{\gamma -\pi }{2}\approx \frac{n-n_{0}}{n_{0}}\frac{\pi -\gamma }{2}.$$

The field intensity distribution of the output Bessel beam is given by

(2) $$I\left(r,z\right)=\frac{4k_{r}P}{w_{c}}\frac{z}{z_{max}}J_{0}^{2}\left(k_{r}r\right)exp\left[-\frac{2z^{2}}{z_{max}^{2}}\right]$$

where $J_{0}$ is the zeroth-order Bessel function of the first kind, P is the power of the incident beam, $w_{c}$ is the beam waist of the beam illuminating the axicon, and r and z are the radial and longitudinal coordinates, respectively. $k_{r}=k\;sin\theta$ and $k_{z}=k\;cos\theta$ are the components of wave vector *k* along r and z, respectively [9]. Finite BB propagation distance $z_{max}$ is defined as

(3) $$z_{max}=\frac{w_{c}}{tan\theta }$$

A Bessel beam described by Equation (2) is expected to have a propagation-invariant core radius $r_{o}$, calculated as

(4) $$r_{o}=\frac{2.405}{ksin\theta }.$$

The factor 2.405 is derived from the first root of the zeroth-order Bessel function [3,15]. This propagation- invariant core is one of the most useful features of Bessel beams, apart from the property of being able to self-heal after encountering an obstruction. The reconstruction capability of BBs is attributed to the wave vectors propagating on the surface of a cone. If an obstacle is placed at the center of the beam, the waves that continue propagating can reconstruct the BB beyond the obstruction [3,5,6,7]. Minimum distance $z_{min}$ at which the beam reconstructs itself is

(5) $$z_{min}\approx \frac{b}{2tan\;\theta },$$

where *b* is the width of the obstruction [3,7].

**Figure 2:**
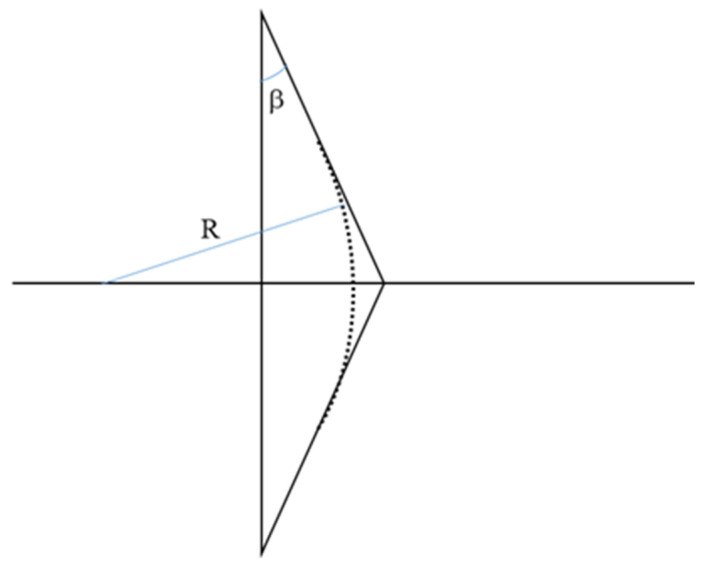
An axicon with a rounded tip described by radius of curvature R and base angle β.

Our theoretical description, up to this point, assumes an ideal axicon with a perfect conical geometry. In practice, the tips of actual axicons are rounded due to fabrication constraints [10,11,12,13,14,15,16,17]. Figure 2 illustrates this rounding of an axicon tip, forming a small lens with a radius of curvature R and a corresponding focal length [20,22]. Refraction by the curved surface modulates the intensity and core diameter of the generated BB as it propagates along *z_max_* [9,16,17,18,19,20,21,22]. The oblate tip focuses part of the incident beam propagating closer to the optical axis, creating a nearly spherical wave after the axicon [9,20,21]. For a perfect conical lens, a Bessel beam should form immediately after the axicon tip, as soon as two conical wavefronts interfere with each other. In the case of a more realistic axicon with a rounded tip, a Bessel beam is generated only beyond the focal length of the small lens [20,22]. The maximum intensity may also be shifted due to partial focusing by the axicon tip [9,16,19,20]. An oblate axicon tip leads to intensity oscillations in the paraxial regime that, in turn, alter the core diameter of BBs. This propagation-dependent core diameter, $d\left(z\right)$, with *z* being the distance measured from the tip of the axicon [19,21], is expressed as

(6) $$d\left(z\right)\approx 2\sqrt{{\left[\frac{M^{2}\lambda }{\pi \left(n-1\right)sin\beta }\right]}^{2}+{\left[az\left(n-1\right)-R\right]}^{2}{sin}^{2}\beta ,}$$

where $M^{2}$ is the propagation parameter of the beam incident on the axicon, β=90°−γ/2 is the base angle of the axicon [19,21], and a is a scaling factor introduced in [21] to accommodate any deviations from the ideal propagation behavior along z that originate from imperfect axicon geometry. Factor a allows $d\left(z\right)$ to assume a constant value for an ideal axicon with R=0 by setting a=0.

Tuning Bessel beam profiles generated by an axicon is not a straightforward task [15], with a solid lens having a constant refractive index and fixed focal length [27,28,29], and a static apex angle [5,15,16,17,18]. Available methods for modifying BB intensity distributions, such as replacing optical elements with alternate lenses or axicons [5,10,15,20,27,28,29,30] and mechanical translation of optical components [21,23,27,28,29,30], have proven to be experimentally tedious, since maintaining correct alignment is critical [3], and poorly scalable [30]. In our experiments, a telescope system is employed to magnify the core diameter of our output BB. A telescope consists of two lenses separated by a distance equivalent to the sum of their focal lengths. Magnification facilitates the measurement of core diameters and, more importantly, extends the maximum propagation distance of BBs [9]. Before passing through the telescope, the axicon-generated Bessel beam undergoes refraction through a fluid chamber.

Fluids have been used to reconfigure optical properties either through the physical deformation of a lens or by making use of a liquid with a variable refractive index [27,28,29,30]. Refractive indices of fluids may be modified using pressure control and electrowetting, as well as optical, magnetic, and thermo-optic excitation [27,28,29,30]. Our present technique applies the most common and simplest method to control the refractive index: filling a refracting chamber with different types of fluids [27]. In [25], a fluid chamber was placed after the focusing lens, with the tuning of propagation properties and core diameters being attributed to a modified focal length of the lens brought about by the refraction of the BB through liquid layers. Our present system makes use of an oblate-tip axicon to generate Bessel beams. BBs then propagate through a refracting chamber, with different combinations of fluid layers, and are expanded by a telescope. For proof-of-concept experiments, we chose to employ a chamber having only two compartments, the simplest design that would allow us to observe the effects of different combinations of fluid refractive indices.

**Figure 3:**
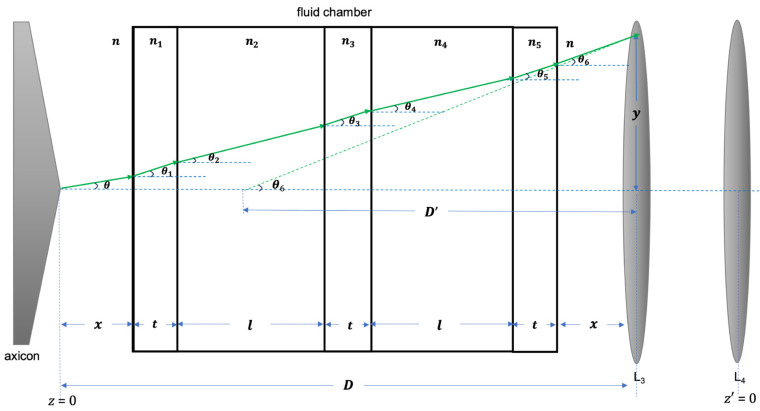
Schematic diagram of a Bessel beam wave vector being deflected through a fluid chamber: axicon–telescope distance *D* is reduced to *D*’ due to refraction through walls of thickness *t* and fluid sections of length *l*.

Let us consider a fluid chamber with two equally sized compartments, placed between the axicon and the first lens of the telescope, as in Figure 3. The surrounding medium is air, with *n* = 1. The refractive indices of the chamber are *n*_1_ (left wall), *n*_2_ (compartment 1 fluid), *n*_3_ (middle wall), *n*_4_ (compartment 2 fluid), and *n*_5_ (right wall). Because of a blunt axicon tip, the Bessel beam profile incident on the lens varies depending on axicon–telescope (a-t) distance D. Our mechanism for manipulating the BB intensity profile relies on refraction through the fluid chamber to alter the optical path length, resulting in a shorter effective a-t distance *D*’.

We use Equation (1) to obtain opening angle *θ* of the output Bessel beam from the axicon. A wave vector traveling on the surface of a cone described by *θ* enters the fluid chamber and is subsequently refracted. Snell’s law is applied at each interface to determine how the cone angle changes. For the left wall,

(7) $$n\sin\theta =n_{1}sin\theta_{1}.$$

Variations of Equation (7) are employed for each combination of refractive indices, yielding modified cone angles *θ*_2_ to *θ*_6_. The deflection of the wave vector by vertical translation *y*, obtained from the respective cone angles and distances *x*, wall thicknesses *t*, and compartment lengths *l*, leads to *θ*_6_ given by

(8) $$tan\theta_{6}=\frac{y}{D^{\prime }}$$

The effective a-t distance *D*’ is then

(9) $$D^{\prime }=\frac{y}{tan\theta_{6}}$$

Before entering the first lens, *L*_3_, of the telescope, the BB core diameter corresponds to $d\left(z\right)$ at *z* = *D*’. The telescope then magnifies the beam profile by a factor *m*. Upon exiting the second lens, *L*_4_, of the telescope system, the propagation-dependent core diameter is described by a modified version of Equation (6):

(10) $$d\left(z^{\prime }\right)\approx 2m\sqrt{{\left[\frac{M^{2}\lambda }{\pi \left(n-1\right)sin\beta }\right]}^{2}+{\left[az^{\prime }\left(n-1\right)-R\right]}^{2}{sin}^{2}\beta }$$

where $z^{\prime }=z-D\prime$. Equation (10) considers the telescope system as a point element on the *z*-axis with the distance between L_3_ and L_4_ being incorporated in the magnification factor *m*. Any pair of lenses with a consistent ratio of focal lengths would produce the same core diameter that changes according to the expression for $d\left(z^{\prime }\right)$, regardless of the actual separation between lenses. Since $D^{\prime }$ changes as a function of refractive indices, the core diameter at a certain distance from the telescope may be varied by using different fluids in compartments 1 and 2 of the chamber. A wide range of BB profiles becomes available using fluids with appropriate refractive indices. In addition, this approach maintains fixed positions for the axicon and telescope, avoiding any misalignment that may arise from adjusting *D* by moving the optical components.

## 3. Materials and Methods

**Figure 4:**
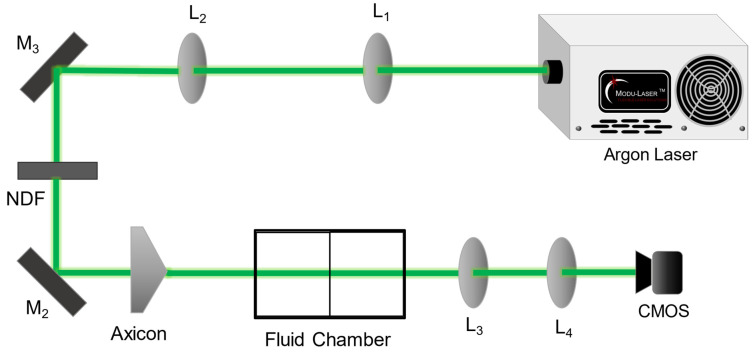
Optical system for generating variable Bessel beams using refraction through a fluid chamber. *L* = lens, *M* = mirror, NDF = neutral density filter.

Our optical set-up for generating zero-order Bessel beams and varying output intensity profiles is illustrated in Figure 4. An incident Gaussian beam from an Ar^+^ laser (Stellar Pro, UT, USA), operating at 25 mW and wavelength *λ* = 514 nm, is collimated by lenses *L*_1_ and *L*_2_ with focal lengths *f*_1_ = 5 cm and *f*_2_ = 50 cm, respectively. The beam is directed by mirrors *M*_1_ and *M*_2_ to an axicon with apex angle γ=178.7° and index of refraction equal to 1.46. A custom-built fluid chamber is placed *x* = 5 cm from the axicon. The chamber walls are window glass (*n*_1_ = *n*_3_ = *n*_5_ = 1.52) of thickness *t* = 3.2 mm. Each fluid compartment has length *l* = 7 cm. Magnification of the Bessel beam is performed with a telescope system with lenses *L*_3_ and *L*_4_ (focal lengths *f*_3_ = 2.5 cm and *f*_4_ = 25 cm, respectively), with L_3_ being positioned *x* = 5 cm after the chamber. Images are captured with a 4.92 MP CMOS camera (Industrial IDS, Mainz, Germany) with sensor dimensions 5.6 mm × 4.2 mm. Appropriate neutral density filters are used to suppress the intensity of the beam to prevent image saturation. The camera is mounted on a track to record Bessel beam intensity profiles at various propagation distances from *z*’ = 7.5 cm to *z*’ = 80 cm, with *L*_4_ at *z*’ = 0.

To minimize aberrations introduced by the fluidic system, our experiments make use of liquids with refractive indices that are comparable to those of the chamber walls (*n* = 1.52) and the axicon (*n* = 1.46). The refracting fluids employed are water (*n* = 1.33), 1000 CST silicone oil (*n* = 1.405), and mineral oil (*n* = 1.48). These liquids are considered safe and free of possible health hazards [23,28]. The selected liquids are also immiscible and exhibit minimal evaporation rates when subjected to increased temperatures, which is an important consideration for high-intensity applications [23,27].

The self-healing of generated BBs modified with different liquid combinations is demonstrated by introducing a copper wire with a diameter of approximately 118 µm as an obstacle. The wire is mounted on a standard microscope slide and placed 2.5 cm after *L*_4_.

## 4. Results and Discussion

**Figure 5:**
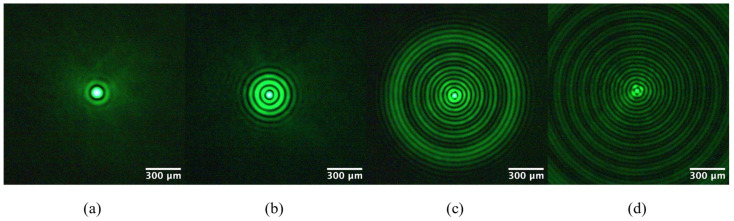
Images of Bessel beams from a blunt-tip axicon at (**a**) *z* = 16 cm, (**b**) *z* = 20 cm, (**c**) *z* = 30 cm, and (**d**) *z* = 40 cm. Core diameters become smaller, while the number of rings increases with the increase in distance.

The images in Figure 5 are intensity cross-sections of zero-order Bessel beams generated by our axicon, without the liquid chamber and without the action of a telescope, captured at arbitrary distances along *z*. A well-defined Bessel beam, having a fully formed first bright ring, is observed to have a core diameter of 117.5 µm at *z* = 16 cm. An ideal axicon should generate Bessel beams immediately after the axicon [9,22]. The core diameter decreases to 72.2 µm at *z* = 20 cm. The central spot continues to become smaller as the beam propagates, with values of 55.8 µm and 49.8 µm at *z* = 30 cm and *z* = 40 cm, respectively. A 31.14% reduction in core diameter is noted for this propagation range of 20 cm. The number of concentric rings is seen to increase with *z*. The variability in the BB core diameter with *z* is consistent with previous results obtained with a blunt-tip axicon [9,19,20,21]. In addition to the reduction in core diameter as the beam propagates, there is an obvious astigmatic transformation of the BB [8], observed at *z* = 40 cm (Figure 5d).

**Figure 6:**
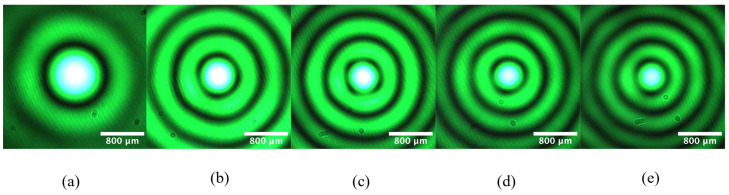
Dependence of output Bessel beams, generated without the liquid chamber, on the distance between axicon and telescope: intensity profiles recorded at *z*’ = 25 cm with (**a**) *D* = 20 cm, (**b**) *D* = 25 cm, (**c**) *D* = 30 cm, (**d**) 35 cm, and (**e**) *D* = 40 cm. Core diameters are observed to decrease with *D*.

The addition of a telescope allows us to change the output BB intensity profile by setting distance D between the axicon and the telescope. The intensity cross-sections in Figure 6 were all captured at *z*’ = 25 cm but were generated with different values of *D*. At *D* = 20 cm, the measured core diameter is 1146.9 µm. Extending *D* to 25 cm leads to a central-spot width of 776.4 µm, a reduction of 32.3%. The core diameters drop to 670.2 µm, 637.8 µm, and 619.7 µm at distances of 30 cm, 35 cm, and 40 cm, respectively. Furthermore, the rings of the generated Bessel beams increase as *D* increases, but the width and intensity of the rings are diminished. Over this 20 cm shift in *D*, the core diameter may be decreased by 46%. The propagation dependence of the transverse intensity profile of Bessel beams is attributed to the roundness of the axicon tip [9,19,20,21,22], along with minor alignment issues caused by the physical translation of the telescope [30]. We note that, with the details of our optical set-up and the geometry of the axicon (*γ* = 178.7°, *n* = 1.46, *λ* = 514 nm, *m* = 10), the theoretical value of Bessel beam radius r_o_ using Equation (4) is 377 µm. The predicted diameter, *d*_0_ = 754 µm, is comparable to the experimental value at *D* = 25 cm. For micromachining applications, reversing the lenses of the telescope to affect demagnification would lead to smaller core diameters, but the maximum propagation distances would be shortened [9]. 

**Figure 7:**
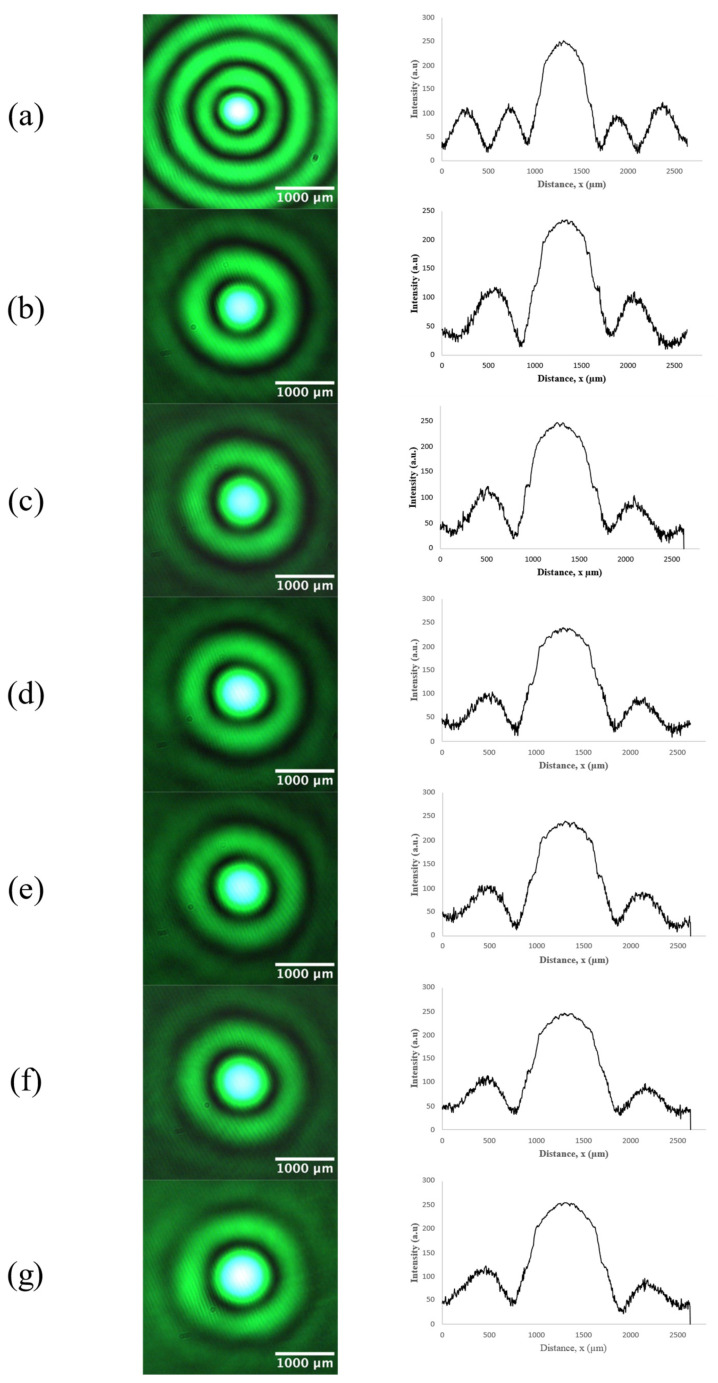
Intensity profiles (left) of Bessel beams are altered using refraction through different liquid combinations: (**a**) empty chamber, (**b**) H_2_O–H_2_O, (**c**) Si oil–H_2_O, (**d**) Mi oil–H_2_O, (**e**) Si oil–Si oil, (**f**) Mi oil–Si oil, and (**g**) Mi oil–Mi oil. Line scans (right) across the centers of the beams show how the core diameter widens as the fluid refractive index becomes higher.

After setting D at 25 cm, we add the fluid chamber to the optical system and apply different combinations of refractive indices to modify the BB intensity profiles. Figure 7 presents intensity cross-sections of BBs refracted by various fluid combinations at *z*’ = 30 cm. For an empty fluid chamber, the core diameter is 773.8 µm. Filling both compartments with water (H_2_O-H_2_O) changes the central-spot diameter to 990 µm, an increase of 28%. The core diameter becomes 1036.5 µm when both partitions are filled with silicone oil (Si oil–Si oil) for an increase of 34%. With mineral oil in both compartments (Mi oil–Mi oil), we measure a core diameter of 1071.7 µm, corresponding to a 38.4% increase. A Si oil–H_2_O combination yields a diameter of 1011 µm (30.6% increase). Mi oil–H_2_O produces a core diameter of 1032.2 µm, while Mi oil–Si oil leads to a diameter of 1054.2 µm, which are increases of 33.4% and 36.2%, respectively. Of all fluid combinations, the H_2_O–H_2_O combination yielded the smallest increase in core diameter, while the Mi oil–Mi oil combination produced the largest. Fluids with higher indices of refraction result in a shorter effective axicon–telescope distance *D*’, consistent with our analysis based on Equations (9) and (10). As previously discussed, decreasing the gap between axicon and telescope leads to larger central-spot widths. These results are in agreement with [21], where moving a telescope system closer to the axicon created BBs of wider core diameters. No physical movement of optical components is required for our approach involving refraction through liquid media.

Table 1 summarizes the fluid parameters and core diameter results for all combinations. Theoretical values for *D*’ and d(*z’*) are calculated using Equations (9) and (10), respectively, with Equation (10) using the parameters ${\;M}^{2}=1$, n=1.46, β=0.65°, R=0.05 cm, *m* = 10, and a=0.035. Scaling factor a is consistent with the fitting parameter reported in [21]. As expected, due to symmetry in refraction behavior, the measured core diameters for H_2_O–Si oil (1012.5 µm) and Si oil–H_2_O (1011 µm) are almost equal because of similar values of *D*’, as per Equation (9). Similar symmetry is found for the fluid pairs H_2_O–Mi oil (1032.2 µm)/Mi oil–H_2_O (1032.2 µm) and Mi oil–Si oil (1054.2 µm)/Si oil–Mi oil (1053.2 µm). In general, our theoretical framework, based on an effective axicon–telescope distance brought about by refraction through the fluid chamber, accurately describes the experimental variation in the BB core diameter.

**Figure 8:**
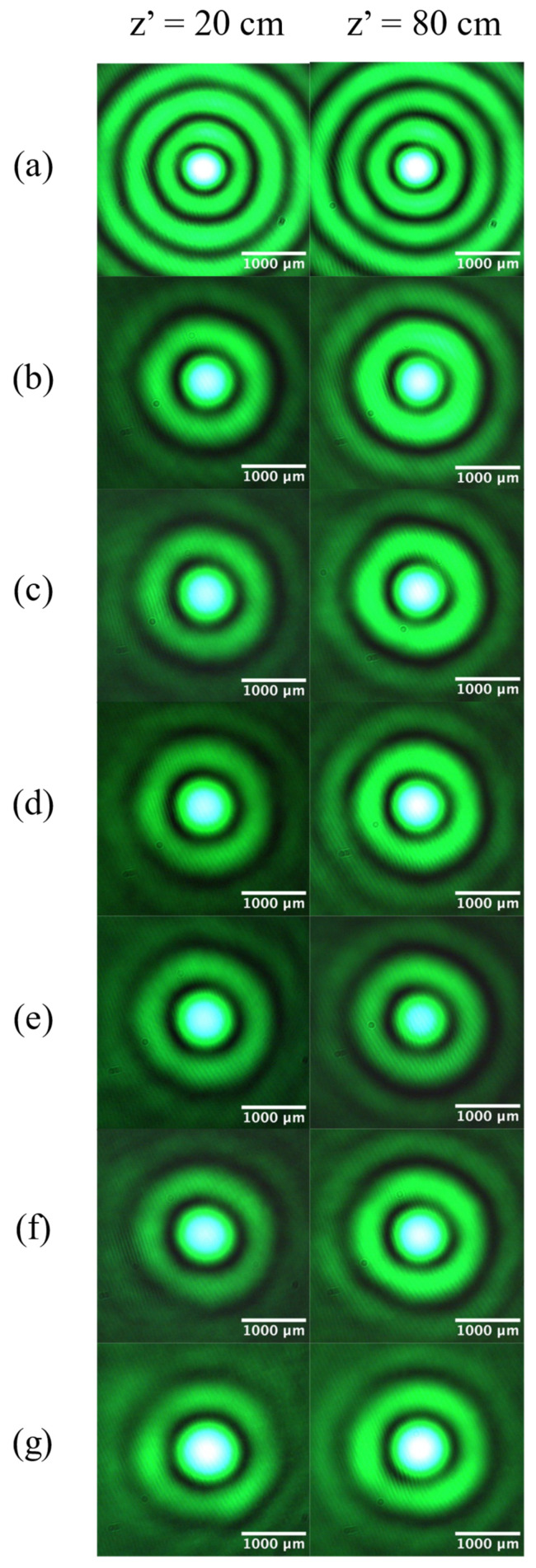
Images of Bessel beams at *z*’ = 20 cm and *z*’ = 80 cm showing propagation invariance: (**a**) empty chamber, (**b**) H_2_O–H_2_O, (**c**) Si oil–H_2_O, (**d**) H_2_O–Mi oil, (**e**) Si oil–Si oil, (**f**) Si oil–Mi oil, and (**g**) Mi oil–Mi oil.

Although our optical system exploits the distance-dependent propagation of Bessel beams from an axicon, BBs traveling through the fluid chamber and telescope still exhibit minimal diffraction. Figure 8 compares BB intensity profiles generated with different fluid combinations at *z*’ = 20 cm and *z*’ = 80 cm. A BB passing through an empty chamber has a core diameter of 775.25 µm at *z*’ = 20 cm, which decreases slightly to 764.5 µm at *z*’ = 80, corresponding to a reduction of only 1.4%. Over the same propagation range, the core diameter for H_2_O–H_2_O drops by 7.5%, from 994.75 µm to 920.25 µm. For Si oil–Si oil, the measured core diameter changes from 1054 µm to 991.2, a 5.95% decrease. The Mi oil–Mi oil combination exhibits a core diameter reduction of 3.5%, from 1078.5 µm to 1040.5 µm. Compared with the results in Figure 6 and Figure 7, the variability in the core diameter as the beam propagates for a distance of 60 cm is significantly smaller compared with central-spot effects due to adjusting *D* or refraction through the fluid chamber. Along with this improved non-diffracting behavior, BBs passing through different liquid combinations do not exhibit astigmatic aberration over the observed propagation range.

**Figure 9:**
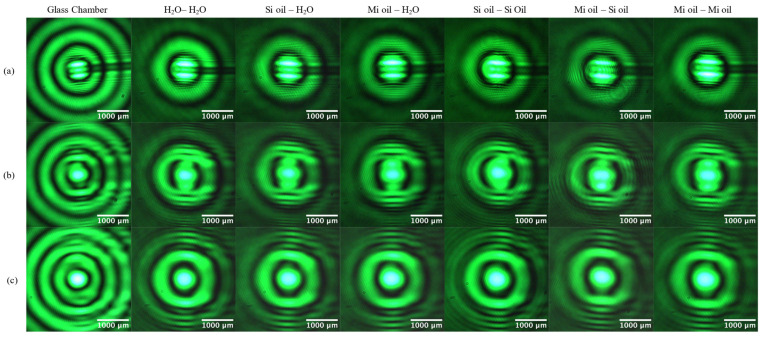
The self-healing of Bessel beams refracted by different liquid combinations, with reconstructed intensity profiles at (**a**) *z*’ = 7.5 cm, (**b**) *z*’ = 30 cm, and (**c**) *z*’ = 60 cm. A wire obstruction with a diameter of 118 µm is placed at *z*’ = 5 cm.

The self-healing of Bessel beams modified using fluid refraction is seen in Figure 9. The copper wire obstruction is visible as a shadow across all intensity profiles at *z*’ = 7.5 cm with recovery of the core already evident at *z*’ = 30 cm. The restoration of the central spot and of the first bright ring is achieved at a propagation distance of 60 cm. BBs propagating through different fluid combinations maintain the ability to recover their profiles after encountering an obstacle. Due to the conical nature of the Bessel beam wave vectors, unobstructed intensity regions contribute to reconstructing the intensity profile [3,5,6,7,20]. Analyzing the effect of the refractive index on the self-healing behavior of Bessel beams passing through a fluid chamber is left for future study.

## 5. Discussion

**Figure 10:**
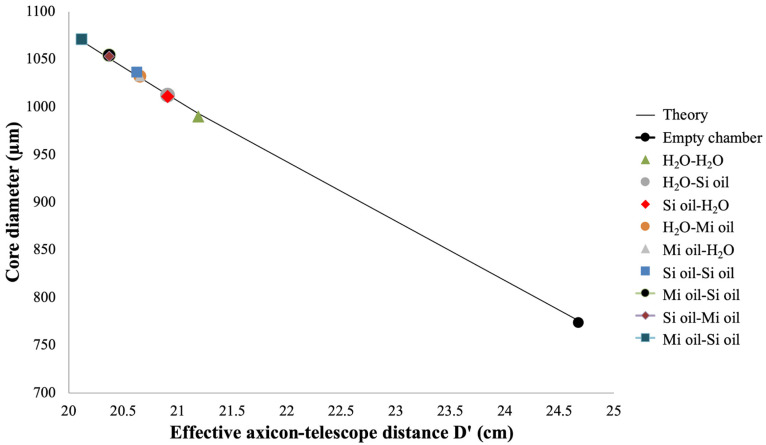
Dependence of Bessel beam core diameter on effective axicon–telescope distance due to different fluid refractive indices.

Further verification of our theoretical analysis is provided in Figure 10, a plot of experimental core diameter as a function of *D*’ for the liquid combinations employed in our set-up. Data points exhibit an excellent overlap with the expected curve, based on Equation (9) for *D*’ and Equation (10) for d(*z*’), with the following parameters: ${\;M}^{2}=1$, n=1.46, β=0.65°, R=0.05 cm, *m* = 10, and a=0.035. The theoretical curve indicates a core diameter change of 293 µm that is available with our technique, given the appropriate combination of fluids. We reiterate that the effective shift in separation between axicon and telescope is achieved without any actual movement of optical elements.

The propagation invariance of BBs refracted by liquids may be enhanced with the selection of fluids with refractive indices similar to that of the chamber walls. We note the largest decrease (8.3%) in core diameter for H_2_O–Si oil and the lowest decrease for Mi oil–Mi oil (3.5%) over a propagation distance of 60 cm. Appreciable differences among the indices of refraction of the fluid chamber walls (*n* = 1.53), axicon (*n* = 1.48), mineral oil (*n* = 1.48), silicone oil (*n* = 1.405), and water (*n* = 1.33) cause reflection and scattering at each interface [21,22,28]. Minimizing differences in refractive indices would keep the BB core diameter fixed over a longer propagation distance. The choice of fluids to be used for Bessel beam modification ultimately depends on the core diameter needed and requirements for non-diffraction.

## 6. Conclusions

In summary, we report the successful design and implementation of a fluid-based system for varying core diameters and intensity profiles of zero-order Bessel beams. Core diameters of BBs refracted by liquids become wider, from 773.8 µm with an empty fluid chamber to 1071.7 µm with two layers of mineral oil. Refracted BBs retain the property of non-diffraction, with a maximum change in core diameter of only 7.5% over a propagation range of 60 cm. The self-healing of the beams is also observed with all fluid combinations. Our results are in excellent agreement with a theoretical framework that combines the propagation-dependent output of a blunt-tip axicon and an effective shift in axicon position achieved with the refraction of a Bessel beam through liquids.

The ability to resize BB core diameters using refraction by different fluid combinations finds immediate utility in many applications, such as grooving, drilling, and dicing with high-power lasers. Refraction through fluids has the additional advantage of suppressing side lobes in the active beam spot to improve uniformity in materials processing. Our straightforward technique works with a static fluid chamber and avoids any misalignment issues connected with physical displacement of optical elements. Future investigations may consider chamber designs with additional compartments for generating an even wider range of Bessel beam profiles. Measurements of fluid refractive indices should be possible with proper calibration of the system.

## Figures and Tables

**Table 1:** Summary of parameters and core diameters for all fluid combinations at *z*’ = 30 cm.

Fluid Combination	Chamber 1 Refractive Index	Chamber 2 Refractive Index	Theoretical *D*’ (cm)	Theoretical Core Diameter d(*z*’) (µm)	Experimental Core Diameter (µm)
Empty chamber	1	1	24.7	776	773.8
H_2_O–H_2_O	1.33	1.33	21.2	993.1737	990
H_2_O–Si oil	1.33	1.405	20.9	1012.9353	1012.5
Si oil–H_2_O	1.405	1.33	20.9	1012.9353	1011
H2O–Mi oil	1.33	1.48	20.7	1030.8974	1032.2
Mi oil–H_2_O	1.48	1.33	20.7	1030.8974	1032.2
Si oil–Si oil	1.405	1.405	20.6	1032.9396	1036.5
Mi oil–Si oil	1.48	1.405	20.4	1051.1119	1054.2
Si oil–Mi oil	1.405	1.48	20.4	1051.1119	1053.2
Mi oil–Mi oil	1.48	1.48	20.1	1069.4597	1071.2

## Data Availability

The data used in this study is available upon request from the corresponding author.
